# MALDI-TOF Mass Spectrometry for Glioblastoma Secretome Biomarker Screening: A Review of Challenges and Perspectives

**DOI:** 10.3390/cimb48060627

**Published:** 2026-06-16

**Authors:** David Aebisher, Klaudia Dynarowicz, Rostyslav Marunych, Izabela Rudy, Kacper Rogóż, Aleksandra Kawczyk-Krupka, Piotr Oleś, Dorota Bartusik-Aebisher

**Affiliations:** 1 Department of Photomedicine and Physical Chemistry, Faculty of Medicine, University of Rzeszów, 35-959 Rzeszów, Poland; 2 Department of Biochemistry and General Chemistry, Faculty of Medicine, University of Rzeszów, 35-959 Rzeszów, Polanddbartusikaebisher@ur.edu.pl (D.B.-A.); 3 Doctoral School, Faculty of Medicine, University of Rzeszów, 35-310 Rzeszów, Poland; rostyslavm@dokt.ur.edu.pl; 4 Student Scientific Club of Biochemists URCell, Faculty of Medicine, University of Rzeszów, 35-959 Rzeszów, Polandkr117626@stud.ur.edu.pl (K.R.); 5 Department of Internal Diseases, Angiology and Physical Medicine, Center for Laser Diagnostics and Therapy, Medical University of Silesia, Batorego 15, 41-902 Bytom, Poland; akawczyk@sum.edu.pl (A.K.-K.); piotroles@o2.pl (P.O.)

**Keywords:** glioblastoma, secretome, MALDI, MALDI-TOF, proteomics, secreted proteins, cell lines, extracellular vesicles

## Abstract

Glioblastoma (GBM) remains one of the most aggressive malignancies, characterized by profound heterogeneity and a dismal prognosis. While genomic and transcriptomic profiling have provided structural insights, they often fail to capture the dynamic interactions within the tumor microenvironment (TME). Secretome analysis—the study of proteins actively secreted by tumor cells—offers a functional readout of these interactions and a reservoir for potential biomarkers. In this review, we critically evaluate the role of MALDI-TOF Mass Spectrometry as a strategic tool for GBM secretome profiling. While Liquid Chromatography-Tandem Mass Spectrometry (LC-MS/MS) remains the gold standard for deep protein discovery, we argue that MALDI-TOF’s speed, cost-effectiveness, and high-throughput capabilities position it as an ideal platform for clinical screening and “spectral phenotyping.” We discuss the technical hurdles, such as ion suppression and the “leakome” (intracellular contamination), and highlight how integrating MALDI with Extracellular Vesicle (EV) enrichment and Artificial Intelligence (AI) can bridge the gap between in vitro discovery and clinical application.

## 1. Introduction

Studies of the cancer secretome, including glioblastoma (GBM), are increasingly important. They allow us to understand communication between cancer cells and the tumor microenvironment at the molecular level. Secretome profiling involves identifying and quantifying proteins and other factors released by cells. In the context of GBM, mass spectrometry (MS) techniques, such as MALDI (Matrix-Assisted Laser Desorption/Ionization), enable the mapping of proteins from cell culture media. This mapping reveals differences in secretory profiles between lines with varying aggressiveness, invasive potential, or response to treatment [1,2,3]. For instance, label-free quantitative MS has revealed that iHDAC inhibitors can modulate tumor behavior by specifically remodeling the ‘angiogenic matrisome’—regulating factors like decorin and ADAM-family proteases (ADAM10, 12, 15)—rather than through direct VEGF inhibition [4]. This highlights a crucial mechanistic link: histone post-translational modifications (PTMs) regulate chromatin architecture to drive tumor progression, and targeting the enzymes responsible (e.g., deacetylases or methyltransferases) is a rapidly evolving therapeutic strategy [5]. Profiling the secretome serves as a functional readout of this epigenetic state. It allows researchers to monitor how epigenetic therapies remodel the tumor microenvironment. This approach enables the identification of candidate biomarkers. More importantly, it helps researchers understand how therapeutic changes influence cell secretion. These changes, in turn, affect microenvironment remodeling, angiogenesis, invasion, and immune response. In short, secretome profiling is a tool that combines basic research on cellular mechanisms with the search for biomarkers and therapeutic targets [6,7]. This approach allows for the identification of candidate biomarkers, but more importantly, it offers insights into the mechanisms of therapy resistance and relapse, which are the primary clinical challenges in GBM management. For instance, understanding the molecular shifts in Temozolomide (TMZ)-resistant cells is crucial for identifying new therapeutic vulnerabilities. Recent computational studies utilizing gene expression profiles have successfully identified BMS345541—a drug targeting the FOXG1 transcription factor—as a potential repurposed agent to reverse TMZ resistance [5]. Applying secretome profiling in this context could similarly reveal extracellular effectors of such resistance pathways, moving beyond mere description to potential functional intervention. While liquid chromatography-tandem mass spectrometry (LC-MS/MS) remains the gold standard for deep-dive protein discovery due to its high sensitivity, MALDI-TOF MS offers unique advantages for clinical secretome screening. Primarily, MALDI-TOF MS provides unmatched throughput and cost-efficiency, enabling the analysis of hundreds of samples in a fraction of the time required for LC-MS/MS. Furthermore, MALDI-TOF MS is particularly effective in the low-molecular-weight range (peptidomics), capturing bioactive peptide fragments (<10 kDa) that are often lost during the extensive fractionation or enzymatic digestion steps of shotgun proteomics. This allows for the generation of ‘spectral fingerprints’—rapid, diagnostic molecular signatures that can be used for real-time monitoring of tumor progression and therapy response in a clinical setting. In this review, we evaluate the potential of MALDI-TOF MS in GBM secretome profiling, discussing technical constraints, clinical translation challenges, and future perspectives.

## 2. Glioblastoma Pathophysiology and Research Models

Glioblastoma (GBM) is defined by its profound intra- and intertumoral heterogeneity, manifesting across genomic, transcriptomic, and epigenetic layers [8,9,10,11,12]. While clinical management relies on molecular subtyping—including the assessment of isocitrate dehydrogenase (IDH) mutations, which serve as a primary diagnostic and prognostic indicator, and O-6-methylguanine-DNA methyltransferase (MGMT) promoter methylation, a key predictor of sensitivity to alkylating chemotherapy—and transcriptional classification (proneural, mesenchymal, classical), these static markers often fail to capture the dynamic nature of tumor progression [10,11]. The primary clinical “pain point”—resistance to Temozolomide and inevitable relapse—is driven by a subset of cells that actively remodel their niche [8,11]. In this context, the secretome acts as a critical functional interface; tumor cells modulate the extracellular space to promote invasion, angiogenesis, and immune evasion [10,11]. Consequently, profiling secreted factors is a necessity to understand how GBM clones adapt to therapy and bypass the limitations of the blood–brain barrier [12,13]. Recent meta-analyses of whole-proteome datasets have identified a core set of dysregulated proteins in GBM, including 154 commonly upregulated and 116 downregulated factors compared to normal brain tissue. These shifts reflect critical alterations in mRNA splicing and immune system pathways, providing a systemic backdrop against which the secretome—as a functional readout of these changes—must be interpreted [14].

In vitro models serve as essential discovery platforms for MALDI-based secretomics, offering high reproducibility and simplified sample preparation required for high-throughput methodological optimization [15,16,17]. These controlled systems allow for the precise mapping of secretome shifts under specific stressors—such as epigenetic modulation or drug exposure—without the systemic noise inherent in in vivo environments [18,19]. However, the translational utility of traditional 2D cultures is limited by the absence of the glioblastoma microenvironment’s architectural complexity, oxygen gradients, and multi-cellular interactions (e.g., with immune and endothelial cells), which can lead to the loss of original tumor characteristics over long passages [8,11,18]. Consequently, while GBM cell lines are indispensable for the initial high-throughput screening of secretome signatures and the development of MALDI-TOF MS workflows, they must be viewed as a foundational step. Validating these in vitro “fingerprints” in more complex patient-derived 3D models (organoids) and longitudinal clinical samples is mandatory to ensure biological relevance and clinical applicability [11,18]. The transition from isolated cell line secretomes to “regional proteomes”—analyzing proteins directly from tumor margins—is the necessary next step. Cell lines serve as a discovery platform where MALDI signatures are first identified under simplified conditions before being validated against the “noisy” background of clinical samples.

## 3. The Secretome as a Diagnostic and Functional Hub

The cancer secretome serves as a dynamic signaling hub that governs the complex cross-talk between GBM cells and the tumor microenvironment (TME) [18,20,21]. Unlike whole-cell proteomics, secretome analysis provides a direct readout of the active mediators of niche remodeling—such as proteases, cytokines, and extracellular vesicles (EVs)—which are potentially detectable in clinical biofluids like plasma and cerebrospinal fluid (CSF) [7,11,18]. It is essential to distinguish soluble secretome components from EV-associated cargo, as they require distinct isolation workflows (e.g., ultrafiltration versus ultracentrifugation) [22,23,24]. Furthermore, while soluble proteins are often analyzed directly, EVs necessitate a specific lysis step to release encapsulated proteins for effective MALDI-TOF MS detection [25,26,27].

In GBM, these secreted factors are primary determinants of invasive growth. Specifically, the enrichment of metalloproteinases and adhesion factors in the extracellular space helps differentiate high-invasive from low-invasive cellular phenotypes [7]. Furthermore, secreted components and exosomal cargo act as paracrine effectors that reprogram surrounding microglia and immune cells, fostering an immunosuppressive environment that facilitates tumor progression and escape [18,28].

However, the identification of a secreted protein is merely a hypothesis-generating step. To move toward clinical utility, these findings must undergo rigorous functional validation, linking candidate markers to core oncogenic drivers, such as the PI3K/AKT signaling axis, or to established mechanisms of TMZ resistance [19,28]. In glioblastoma, oncogenic PI3K/AKT/mTOR signaling downstream of EGFR amplification or PTEN loss not only supports proliferation and therapy resistance but also enhances angiogenic output by increasing VEGF expression and secretion, thereby reshaping the secretome toward a pro-vascular phenotype [29,30,31,32]. In parallel, constitutively active NF-κB orchestrates a chronic inflammatory program in GBM cells, where TNF-α-driven NF-κB activation induces robust IL-6 production, sustains STAT3 signaling, and promotes expression of additional inflammatory mediators, collectively fostering an immunosuppressive, tumor-supportive microenvironment enriched in pro-inflammatory cytokines [33,34,35]. MORE. Moreover, malignant cells display elevated Ca^2+^ signaling dynamics, in which store depletion, store-operated Ca^2+^ entry and calpain activity drive extracellular vesicle (EV) biogenesis; blocking cytosolic Ca^2+^ with chelators such as BAPTA-AM effectively abolishes EV release, underscoring the reliance of vesiculation and docking on Ca^2+^-dependent pathways [35,36,37].

Together, these signaling axes converge to dictate the final composition of the GBM secretome—combining high levels of VEGF and MMP-associated invasive programs, NF-κB-dependent cytokines such as IL-6, and Ca^2+^-regulated EV cargo—to optimally support angiogenesis, invasion, immune evasion and therapeutic resistance.

## 4. Methodological Workflows in GBM Secretomics

Mass spectrometry (MS) is a fundamental tool in proteomics and secretome studies. It enables the identification and quantification of proteins released by cells. Various MS techniques differ in ionization methods, analyzers, and sample preparation. Consequently, the choice of method depends on the study objective and the required level of details [38].

### 4.1. Methodological Landscape: MALDI-MS and LC-MS in Secretomics

The selection of a mass spectrometry platform involves a trade-off between analytical depth and throughput. Direct MALDI-based workflows may involve spectral profiling (“fingerprinting”), peptide mass fingerprinting (PMF), or MALDI imaging mass spectrometry (MALDI-MSI), depending on the analytical objective. Spectral profiling focuses primarily on reproducible signal patterns and biomarker signatures, whereas PMF relies on peptide mass matching for protein identification. This provides a cost-effective method for screening many samples with minimal preparation [39,40]. However, MALDI is inherently limited by ion suppression in complex mixtures; high-abundance proteins (e.g., albumin fragments) often mask low-concentration signaling factors. While MALDI-TOF/TOF MS analysis enables sequence confirmation of selected precursor ions and rapid spectral characterization, direct MALDI-based workflows generally provide lower proteome coverage than LC-coupled tandem MS approaches due to the absence of prior chromatographic peptide separation [41]. In contrast to direct MALDI profiling approaches, LC-coupled tandem MS workflows provide substantially greater analytical depth by separating peptide mixtures prior to ionization. These workflows may utilize either electrospray ionization (LC-ESI-MS/MS) or MALDI-based detection (LC-MALDI-TOF/TOF). By reducing ion suppression and increasing peptide coverage, LC-coupled strategies enable the identification of low-abundance proteins and post-translational modifications (PTMs) [42]. Quantitative approaches such as SILAC, TMT, or iTRAQ further enhance comparative proteomic analyses [43,44,45].

A unique advantage of the MALDI platform is its extension into MALDI Mass Spectrometry Imaging (MSI). MSI bridges the gap between secretomics and histology by mapping the spatial distribution of molecular signals directly on tissue sections without the need for labels [2,46]. In glioblastoma research, this provides critical spatial context. Researchers can correlate protein localization with tumor boundaries and necrotic regions. This information is typically lost in bulk LC-MS/MS analyses [47,48].

### 4.2. Critical Comparison of MALDI and LC-MS Platforms

The choice between MALDI and LC-MS/MS is dictated by the specific requirements of the GBM study—whether the priority is deep discovery of signaling pathways or high-throughput screening of biomarker signatures. The Table 1 summarizes the technical trade-offs between these two dominant platforms.

MALDI-TOF MS is not a replacement for LC-MS/MS in de novo biomarker discovery. LC-MS/MS remains the superior method for depth. Instead, the value of MALDI lies in its role as a clinical screening platform. Once discovery proteomics establishes a “hit list” of proteins, MALDI-TOF MS provides the speed and cost-efficiency to monitor these signatures. This allows for the analysis of large patient cohorts, which is often unfeasible for LC-MS/MS in routine clinical settings [54,58].

## 5. Technical Constraints and Strategic Solutions in MALDI-TOF MS Secretomics

MALDI-based approaches have been applied to glioblastoma tissues, cyst fluids, and model systems. These studies highlight technical constraints and offer strategies for optimization across sample preparation, acquisition, and bioinformatics [2,59]. Such insights are directly adaptable to GBM secretome-focused workflows.

### 5.1. Critical Parameters in Sample Preparation

Matrix and washing: Tissue and cell studies emphasize that washing must remove salts and contaminants without delocalizing analytes. Matrix coating must be homogeneous to preserve spatial and intensity information [15].

Secreted fluids: For GBM cyst fluid, optimized protocols (including weak cation exchange enrichment) were required to reproducibly detect low-mass secreted proteins around 6–7 kDa [60].

Low-volume preparation: For high-resolution MSI and single-cell work, mild fixation with isotonic washes balances morphology and molecular integrity. However, researchers must control for the leakage of cytosolic components to avoid artifacts [15,61].

### 5.2. Strategic Optimization for the GBM Secretome

Optimizing GBM secretome analysis involves three goals: (1) extracting low-abundance proteins like EV cargo, (2) linking them to spatial niches, and (3) monitoring therapy-induced changes (Table 2). The following strategies address these objectives.

EV-focused proteomics: Bottom-up MS of GBM-EVs identifies thousands of proteins. These include invasion-linked markers and post-translational modifications that bulk assays often miss [61,62].

Source optimization: Conditioned media provide EVs highly enriched for tumor material. This improves the detection of low-abundance factors compared to blood samples [63].

Secretome fractionation: Separating EV and EV-depleted fractions reveals distinct proteomes. Both fractions contribute differently to immune and microenvironmental responses [64].

### 5.3. Signal Acquisition and the “Single Peptide” Identification Crisis

MALDI-TOF MS often yields a limited mass range and overlapping peaks, which complicates protein assignment [60]. To mitigate this, high-resolution MALDI-FTICR MSI can be paired with region-specific LC-MS/MS. This alignment reduces false positives by matching accurate masses with localized bottom-up identifications [2]. Furthermore, single-peptide identifications are prone to error. Specificity can be increased using stable isotope-based amino acid mass tagging. This technique allows for higher-confidence calls, particularly for low-abundance or membrane proteins [65].

### 5.4. Bioinformatics and Functional Interpretation of MALDI Data

GBM secretomics workflows can utilize existing bioinformatics pipelines to ensure data quality:

- Database search: Engines like Sequest should use stringent cutoffs, including an FDR <1% and a requirement for at least two unique peptides [66].
- Functional annotation: Analysis should focus on proteins linked to the angiogenic matrisome and GBM progression [67]. Using dedicated EV databases, such as Vesiclepedia [Available online: http://www.microvesicles.org/ (accessed on 01 June 2026)], helps contextualize tumor specificity [68].
- Clinical correlation: Linking MALDI patterns with patient outcomes demonstrates the feasibility of actionable biomarker panels [2,59].

**Figure 1:**
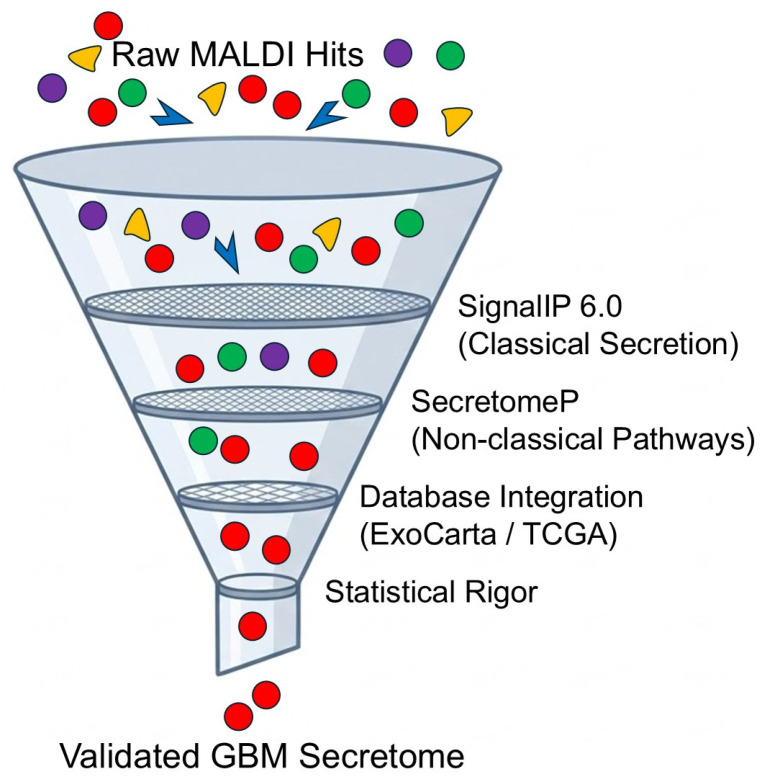
Multi-stage bioinformatic pipeline for the high-confidence identification of the glioblastoma secretome. To ensure the biological relevance of MALDI-TOF MS profiles, raw protein hits undergo a rigorous filtration process to discriminate the authentic secretome from intracellular contaminants (leakome). Stage 1: Identification of classical secretion signals via SignalP 6.0. Stage 2: Prediction of non-classical (leaderless) secretion pathways using SecretomeP 2.0, critical for tumor stress response. Stage 3: Cross-referencing with extracellular vesicle (EV) and cancer repositories (e.g., ExoCarta, Vesiclepedia, TCGA) to contextualize protein origin and clinical relevance. Stage 4: Application of stringent statistical thresholds (FDR < 1% and minimum 2 unique peptides) to yield a validated set of clinically actionable GBM biomarkers. Note: The diagram represents the bioinformatic filtration pipeline following the initial sample preparation and EV isolation (created by the authors).

A multi-stage pipeline for GBM MALDI secretomics (Figure 1) should therefore: (1) distinguish classical from non-classical secretion signals [47]; (2) integrate results with prognostic datasets like TCGA [67,68,69,70]; and (3) apply strict statistical thresholds to define a refined secretome signature.

In summary, core MALDI constraints are mitigated through careful sample enrichment, high-resolution mass spectrometry, and FDR-controlled bioinformatics. When integrated with clinical data, these workflows transform fragile peptide hits into robust biomarker panels.

## 6. Synthesis of Published Research: From Deep Discovery to MALDI Targets

The synthesis of published research highlights a strategic pipeline: while deep discovery via LC-MS/MS has provided a comprehensive atlas of the GBM secretome, these findings now serve as the foundational ‘hit list’ for rapid MALDI-TOF MS clinical screening. The following sections evaluate these studies not as competing methodologies, but as complementary stages in biomarker development.

### 6.1. The “Core” GBM Secretome: High-Abundance MALDI Candidates

Despite varying methodologies, a subset of high-abundance proteins—primarily extracellular matrix (ECM) components—emerges in almost every study. Proteins such as Tenascin-C (TNC), Fibronectin (FN1), and SPARC are not only biologically significant as mediators of tumor stiffness and invasion but are also ideal targets for MALDI-TOF MS profiling due to their high concentration in conditioned media [71,72,73]. These proteins are not only central to GBM invasiveness but also yield high-intensity, reproducible peaks in MALDI spectra. Additionally, secreted factors like YKL-40 and Osteopontin, frequently dysregulated in aggressive GBM phenotypes, serve as robust ‘sentinel’ markers that can be monitored via MALDI-TOF MS to track the transition from low- to high-invasive states [74].

### 6.2. Phenotype-Specific Markers and Methodological Discrepancies

The reported discrepancies in protein counts between studies (ranging from 150 to nearly 1000) are primarily a reflection of the analytical platform used. For instance, proteins such as YKL-40, identified in invasive GBM phenotypes through MS/MS discovery workflows [6], could serve as candidate targets for future MALDI-based profiling strategies aimed at translational biomarker development. In contrast, patient-derived sphere models (GICs) secrete unique factors like Midkine (MDK) [75]. The fact that MDK is often missed in serum-cultured lines underscores the need for MALDI protocols that can operate in serum-free, chemically defined environments to capture these low-abundance stemness mediators. Table 3 shows a review of studies analyzing the secretome of the GBM lines.

### 6.3. Bridging the Gap: Why MALDI Matters for These Findings

**Figure 2:**
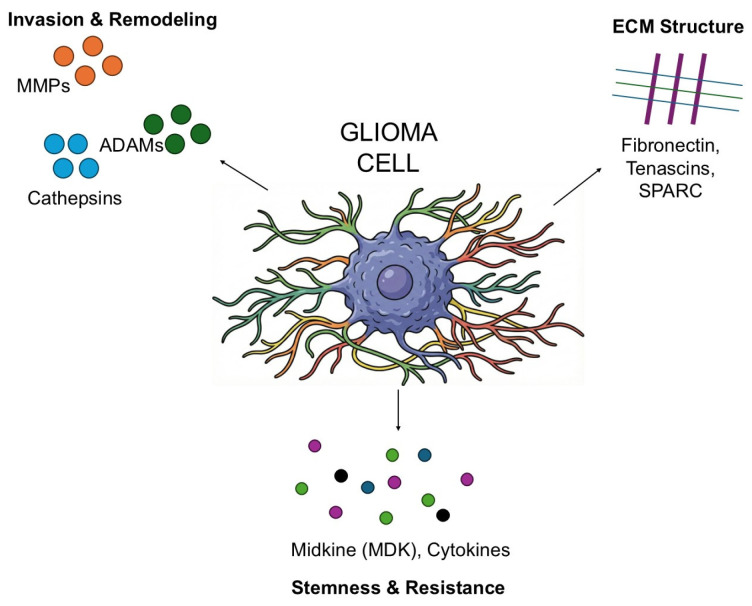
Proposed Key components of the Glioblastoma Secretome identified by MS proteomics. The secretome acts as a functional interface driven by core ECM proteins (e.g., Tenascins), invasion mediators (Proteases, ADAMs), and stem-cell specific factors (e.g., Midkine). These secreted molecules drive microenvironmental remodeling, therapy resistance, and tissue invasion (created by the authors).

While deep LC-MS/MS by Gupta et al. [71] identified nearly 1000 proteins, many of these are low-abundance intracellular contaminants. For clinical translation, we do not need to detect every protein; we need to monitor the “secretory signature.” The predominant ECM and invasion proteins identified in these studies (Table 4) produce distinct, high-intensity ions in MALDI spectra. This confirms that the transition from LC-MS/MS “discovery” to MALDI-TOF MS “screening” is not only possible but biologically justified. Figure 2 shows proposed Key components of the Glioblastoma Secretome identified by MS proteomics.

### 6.4. Analytical Constraints of MALDI-TOF MS in Secretomics: From Quantitative Performance to Potential Failure Modes

In contrast to LC–ESI–MS/MS, MALDI-TOF(/TOF) typically offers moderate resolution (~10,000 or higher in reflector mode) with picomole–femtomole peptide sensitivity but suffers from limited quantitative robustness and lower analytical sensitivity in complex clinical samples, where it cannot reliably detect low analyte loads without enrichment [38,53,78]. Quantitative MS/MS comparisons using iTRAQ-labeled E. coli digests show that LC-MALDI-TOF/TOF and LC-ESI-QTOF yield very similar peptide ratios over 1:1–10:1 ranges, with mean ratio deviations of only 0.7–6.7%, but LC-MALDI uses fewer spectra and achieves slightly lower sequence coverage [53]. However, MALDI-based quantification is intrinsically hampered by poor shot-to-shot and spot-to-spot reproducibility due to heterogeneous matrix–analyte cocrystals and random laser sampling, requiring extensive averaging and internal standards to approximate the quantitative performance routinely achieved by LC–MS/MS [79,80]. In glioma secretome workflows, MALDI-TOF can fail when residual salts or ammonium buffers reduce peptide sensitivity and promote matrix cluster formation that dominates spectra below *m*/*z* 1200, unless carefully optimized desalting and washing (e.g., ammonium phosphate) are applied [78]. Highly abundant ECM proteins and adhesion molecules secreted by glioblastoma cells and EVs (e.g., proteins involved in cell–matrix adhesion, and migration) may dominate the proteomic composition of these samples, potentially limiting the detection of low-abundance chemokines [81,82]. This problem is exacerbated by “leakome” contamination in ex vivo or stressed cultures, where intracellular proteins released from damaged cells add intense background that obscures regulated, low-level secreted factors [83]. Conventional MALDI matrices generate a dense chemical background below ~700–800 *m*/*z*, making it difficult to distinguish low-molecular-weight cytokines from matrix clusters; in an ECM-rich glioma secretome this low-mass interference, combined with ion suppression by abundant matrisome proteins, provides a mechanistic explanation for false-negative MALDI-TOF results for small cytokines, even when LC–MS/MS can detect them [79,80,84].

## 7. Clinical Validation and the Translational “Handover”

The transition from an in vitro secretome “hit list” to a clinically useful biomarker requires a rigorous validation cascade. This process, termed the “Clinical Handover,” involves shifting from broad discovery platforms to targeted, high-sensitivity assays capable of detecting tumor-derived signals against the massive protein background of human biofluids.

### 7.1. From Discovery to Targeted Verification: SRM/PRM vs. Immunoassays

While immunological approaches such as ELISA or conventional IHC remain indispensable for single-target validation, they encounter substantial limitations when confronted with the extreme intratumoral heterogeneity of glioblastoma [85]. The shift away from simple immunological screening toward MALDI-based platforms is therefore not driven by inadequate antibody specificity—indeed, contemporary MALDI-IHC workflows explicitly exploit mass-tagged antibodies [86]. Instead, MALDI’s key advantage lies in its intrinsic multiplexing capacity. Whereas standard immunoassays are typically restricted to 1–5 analytes per run, a single MALDI acquisition can concurrently survey dozens of proteins, glycoforms and lipid species, generating a holistic “spectral fingerprint” that more faithfully captures the evolving molecular landscape of the tumor than any single-antibody assay [3,87].

### 7.2. The Analytical Challenge of Biofluid Matrices: CSF vs. Plasma

Translating secretome markers into clinical samples faces two major hurdles: the Blood–Brain Barrier (BBB) and the dynamic range of protein concentrations.

- Cerebrospinal Fluid (CSF): As the fluid in direct contact with the GBM microenvironment, CSF is enriched with the tumor secretome. However, the concentration of GBM-derived proteins is still several orders of magnitude lower than that of systemic albumin [88,89]. MALDI-based screening in CSF requires advanced pre-fractionation (e.g., immuno-depletion of albumin) to reveal the “hidden” tumor signature.
- Plasma/Serum: The BBB significantly restricts the efflux of large protein markers into the systemic circulation, often rendering secretome candidates undetectable in blood without ultra-sensitive enrichment of extracellular vesicles (EVs) [90,91].

From a translational perspective, detecting these markers in plasma remains a formidable challenge. The blood–brain barrier (BBB) acts as a highly selective filter, ensuring that brain-derived proteins are present in systemic circulation only at extremely low concentrations (picomolar to femtomolar range) [92,93]. Furthermore, the overwhelming abundance of plasma proteins—such as albumin and immunoglobulins—creates a significant ‘ionization noise’ in MALDI-TOF MS, often masking the subtle spectral signatures of glioblastoma-specific factors [94,95].

### 7.3. Standardizing the Pre-Analytical Pipeline

The “Clinical Handover” often fails due to a lack of pre-analytical rigor. Factors such as the time from sample collection to centrifugation, the number of freeze–thaw cycles, and the use of specific protease inhibitors drastically affect the stability of the secretome profile [96,97]. For MALDI-TOF MS to be viable in a clinical setting, a standardized SOP (Standard Operating Procedure) for biofluid handling must be implemented, ensuring that the “molecular fingerprint” recorded in the clinic matches the one discovered in the laboratory.

To address the in vivo relevance, MALDI-based workflows must move toward the analysis of regional proteomes from clinical specimens (e.g., tumor core vs. invasive margin). This approach, though analytically challenging due to tissue heterogeneity, provides a more accurate readout of the active mediators of niche remodeling. By correlating in vitro secretome profiles with spatial data from MALDI-Imaging (MSI) of patient tissues, researchers can bridge the gap between discovery and the biological reality of the GBM microenvironment [3].

## 8. Technical and Methodological Limitations: A Critical Assessment

The inherent complexity of the glioblastoma secretome—characterized by a vast dynamic range of protein concentrations and high levels of glycosylation—presents unique challenges for MALDI-TOF MS analysis. Unlike the relatively “clean” intracellular proteome, the secretome is prone to contamination from culture media additives and cellular debris, which can drastically compromise spectral quality [3].

### 8.1. The Dynamic Range and Ion Suppression Challenge

The primary limitation in MALDI secretomics is the masking effect of high-abundance proteins. A few dominant species (e.g., Albumin fragments or Tenascins) can monopolize the available matrix energy during laser desorption, effectively suppressing the ionization of low-abundance signaling peptides (cytokines, growth factors). This suppression is not merely a matter of instrument sensitivity but a fundamental physicochemical competition within the matrix crystals. To mitigate this, specific strategies for protein depletion and fractionated crystallization must be employed [98,99].

### 8.2. Troubleshooting and Optimization Strategies

To ensure reproducible and high-resolution spectral fingerprints, researchers must systematically address the most common failure modes in MALDI workflows. Table 5 provides a comprehensive guide to identifying and resolving these methodological hurdles.

## 9. Future Research Frontiers: Toward Multimodal and Intelligent Diagnostics

Current evidence suggests a paradigm shift. MALDI-MSI is now being integrated with artificial intelligence and complementary imaging modalities to create multimodal diagnostic platforms. In this framework, AI facilitates the high-throughput translation of complex spectral data into distinct disease phenotypes. Additionally, the synergy of longitudinal datasets enables the reconstruction of a “molecular movie” of therapeutic response. Spatially registered MSI further permits high-resolution “secretomics” by correlating molecular signatures with specific cellular microenvironments.

### 9.1. AI-Driven Spectral Phenotyping

AI models are essential for processing complex MALDI data. For example, single-cell (Table 6) MALDI-MSI lipid profiles were recently combined with MALDI-IHC. This automated recognition model accurately distinguished GBM from neuronal cells using triglycerides and sphingomyelins as key features [3]. Similarly, “dry proteomics” (Table 6) employs unsupervised clustering on lipid and protein MALDI-MSI. This method defines GBM subgroups linked to prognosis and builds robust classification models [110]. Finally, fractal-based feature extraction from MALDI-TOF MS spectra of liquid biopsies has enabled the machine-learning diagnosis of GBM. This shows that even compressed pattern descriptors carry vital diagnostic information [111].

### 9.2. The “Molecular Movie”: Longitudinal Monitoring of Therapy Response

Focusing on MALDI as a screening tool allows for high-frequency, longitudinal monitoring. This ‘molecular movie’ of the secretome allows for the detection of multi-target signatures that single-marker immunological tests would likely miss, providing a more robust framework for real-time therapeutic adjustments [113]. A multimodal platform recently combined MALDI-MSI drug mapping with phosphoproteomics in GBM models. This approach revealed heterogeneous drug distribution and adaptive signaling responses to the inhibitor adavosertib [114].

Furthermore, the use of REIMS during surgery, coupled with MALDI-MSI and LC-MS/MS, enabled survival-time classification based on lipid patterns [59]. In parallel, spatially resolved multi-omics pipelines in GBM models have been used to track metabolic therapy effects, such as arginine depletion. These studies highlight critical differences in response between the tumor core and its rim [115].

### 9.3. Spatial Secretomics: MALDI-MSI and the Peribascular Niche

While not always labeled “secretomics,” several MALDI-centric and multimodal studies interrogate spatial metabolic and proteomic niches in GBM: Ultra-high-resolution MALDI-FTICR MSI with microproteomics in a mouse glioma model delineated tumor-specific proteoforms and metabolic proteins, resolving heterogeneous regions that underlie local functional states [2]. Similarly, lipid and peptide MALDI-MSI in large GBM cohorts revealed spatially distinct microenvironments that correlate with patient survival [110,114].

Advanced MSI techniques, combined with isotope tracing, have also mapped metabolic fluxes in glioma models. This research exposed tumor-specific anabolic activity within the brain ecosystem [116]. Finally, spatial transcriptomics has identified perivascular and perinecrotic niches with distinct inflammatory programs [117,118]. Integrating MALDI-MSI with these datasets will enable true “spatial secretomics,” allowing for the detailed study of macrophage–endothelial–tumor interactions.

Beyond imaging, the integration of single-cell proteomics and microfluidic-based secretome analysis offers a path toward resolving the extreme cellular heterogeneity of GBM. While microfluidics allow for the high-sensitivity capture of secreted factors from minimal cell numbers, single-cell approaches ensure that the ‘average’ secretome signal does not mask rare, highly aggressive clones [119,120]. Furthermore, aligning these protein-level findings with spatial transcriptomics data will enable a complete multi-omic reconstruction of the tumor niche, providing a definitive map of how gene expression translates into the functional, secreted proteome [70,118].

## 10. General Discussion and Conclusions

Secretome profiling represents a critical functional layer in glioblastoma research, capturing the active interface between the tumor and its microenvironment—a layer often missed by static genomic or transcriptomic analyses. However, for MALDI-TOF MS to realize its potential as a diagnostic tool, the field must address the persistent challenges of ion suppression, dynamic range, and pre-analytical variability.

The synthesis of current research confirms that a “hybrid” approach—utilizing LC-MS/MS for deep discovery and MALDI-TOF MS for rapid, high-throughput validation—is the most robust strategy for biomarker development. While cell lines remain indispensable as discovery platforms, the integration of spatial imaging (MSI/IHC) and multi-omic data is necessary to bridge the gap between in vitro findings and clinical reality [121].

While this review focuses on glioblastoma, the potential of MALDI-TOF MS profiling extends to other malignancies. For instance, MALDI-based spectral fingerprinting and imaging are increasingly used to classify breast cancer subtypes, identify diagnostic markers in pancreatic cyst fluids, and map the metabolic heterogeneity of lung and prostate tumors [122,123,124,125,126]. The versatility of MALDI-TOF MS in capturing rapid “molecular signatures” across different solid tumors highlights its broad utility as a scalable clinical platform in oncology.

In conclusion, if methodological standards for sample preparation and bioinformatic filtering (e.g., SignalP, FDR control) are rigorously applied, MALDI-based secretomics will move from an exploratory research tool to a cornerstone of personalized GBM management. The transition from “identifying proteins” to “classifying molecular signatures” via AI-driven MALDI platforms offers a promising route to more precise, functionally relevant diagnostics and therapeutic monitoring [127,128].

## Figures and Tables

**Table 1:** Comparison of MALDI-TOF MS and LC-MS/MS in the Context of Glioblastoma Secretomics.

Analytical Workflow	Representative Methodology	Key Strengths	Main Limitations	References
Direct MALDI profiling/spectral fingerprinting	Direct spotting + MALDI-TOF MS profiling	Very high throughput, rapid spectral phenotyping, low cost	Limited proteome depth, ion suppression, weak low-abundance detection	[49,50]
MALDI Imaging Mass Spectrometry (MALDI-MSI)	Spatially resolved MALDI analysis directly on tissue sections	Spatial molecular mapping, preservation of tumor architecture	Lower identification depth, limited quantitation	[3,51]
LC-MALDI-TOF/TOF workflows	LC fractionation followed by MALDI-TOF/TOF MS analysis	Reduced ion suppression, improved peptide coverage while retaining MALDI robustness	More complex offline workflow, lower throughput	[52,53]
LC-ESI-MS/MS (shotgun proteomics)	NanoLC coupled with Orbitrap/QTOF/LTQ ESI-MS/MS	Highest proteome depth, PTM analysis, quantitative workflows	Longer runtime, higher cost and complexity	[54,55]
Targeted LC-MS workflows (SRM/MRM/PRM)	Targeted peptide quantification after discovery phase	High analytical specificity and reproducibility	Requires predefined targets	[56,57]

**Table 2:** Key optimization levers for GBM MALDI secretomics.

Goal	Strategy	Source
**Enrich low-abundance secreted/EV proteins**	Fractionation, cation-exchange, EV isolation	[23]
**Retain spatial/phenotypic context**	Combine MALDI-MSI with IHC or spatial transcriptomics	[61]
**Therapy-response secretome profiling**	High-throughput MS of conditioned media under drug treatment	[59]

**Table 3:** Overview of studies analyzing the secretome of GBM lines.

Author (Year)	Cell Line	Preparation Method	MS Technique	Number of Identified Proteins/Signals	Important Biomarkers/Highlighted Proteins	Validation
**Formolo et al., 2011** [6]	U87, U118, LN18, T98 (four comparison lines)	SILAC on conditional medium (SILAC pair comparisons)	MS/MS (SILAC-labeling, qualitative/quantitative)	Number of differentially secreted proteins reported in comparative analyses; list of significant differentially secreted proteins	ADAM9, ADAM10, cathepsin B, cathepsin L1, osteopontin (SPP1), neuropilin-1, semaphorin-7A, CH3L1 (YKL-40)	Functional testing (CH3L1 antibody inhibition reduced invasiveness by ~30%)
**Polisetty et al., 2011** [76]	HNGC2, LN229, U87MG	Conditioned medium, prefractionation (SDS-PAGE)	LC-ESI-MS/MS (LTQ)	102 (HNGC2), 119 (LN229), 64 (U87MG); 148 non-redundant total	Vinculin, Tenascin-XB, SERPINF1, TIMP-1 (and other ECM and proteases)	List of identifications; comparison to databases; indicated proteins detectable in plasma/CSF → further validation recommended (authors indicated biomarker potential)
**Gupta et al., 2013** [71]	HNGC-2 (GBM model)	Prefractionation, SDS-PAGE from conditioned medium	LC-MS/MS (ESI-IT, LTQ)	996 protein identifications (≥2 peptides)	Functional groups related to DNA repair, cellular organization; possible candidates for clinical trials	Mapping to the transcriptome; authors discuss further validation (WB/ELISA) for selected candidates
**Han et al., 2019** [77]	patient-derived GBM tumor spheres (GICs)	conditioned medium with reduced growth factors (spheres)	shotgun proteomics (LC-MS/MS) + proteomic analyses	a set of secreted proteins characteristic of GICs; selected molecules (MDK)	Midkine (MDK) highlighted as a potential autocrine factor; PCBP4 indicated as a resistance predictor	Extensive validation: immunohistochemistry, Western blot, functional MDK inhibition assays (cytotoxic effect/apoptosis); prognostic analysis on TCGA
**Pancholi et al., 2025** [21]	U87MG (iHDAC effect study)	label-free preparation of secretome from conditioned medium after iHDAC application	LC-MS/MS (label-free quantitative proteomics)	set of ECM proteins and proteases showing significant changes in iHDAC; (quantitative analysis—number of differentially regulated proteins in text)	decorin (DCN), ADAM10, ADAM12, ADAM15 and other components of the “angiogenic matrisome” shown to be regulated by iHDAC	Bioinformatic confirmation; the authors point out the need for further validation and functional studies

**Table 4:** Comparative overview of key protein identifications in GBM secretome studies.

Study	Key Identified Proteins (Examples)	Biological Context	Reference
**Polisetty et al.**	Tenascin-XB, Fibronectin, SPARC, TIMP-1	Core ECM & Matrix Remodeling	[76]
**Gupta et al.**	PI3K/AKT pathway proteins, EGFR mediators	Intracellular Signaling (Deep Profiling)	[71]
**Formolo et al.**	YKL-40 (CHI3L1), ADAM10, ADAM9	Invasion & Inflammation	[6]
**Han et al.**	Midkine (MDK), Pleiotrophin	Stemness & Therapy Resistance	[77]

**Table 5:** Troubleshooting and Optimization Guide for MALDI-based GBM Secretome Profiling.

Common Issue	Probable Source	Recommended Optimization
High background/salt adducts	Residual salts, buffers, or media supplements	Implement desalting or reversed-phase cleanup prior to MALDI analysis to reduce ion suppression and salt clustering [100,101]
Dominant albumin or serum-derived signals	Incomplete serum depletion or ECM-associated serum retention	Extend serum-free adaptation and PBS washing steps; consider depletion or fractionation strategies when compatible with downstream analysis [102,103]
Low signal intensity	Low analyte concentration or suboptimal matrix crystallization	Optimize protein concentration and matrix-to-analyte ratio; evaluate alternative matrices and spotting conditions [104]
Poor low-mass peptide detection	Ion suppression by abundant proteins or peptide loss during filtration	Use matrices optimized for low-molecular-weight analytes and adapt peptide enrichment/fractionation workflows accordingly [105,106]
Limited proteome coverage	High sample complexity without prefractionation	Introduce LC separation, fractionation, or peptide enrichment prior to LC-MS analysis [38,107].
Poor spectral reproducibility	Heterogeneous crystallization or inconsistent spotting	Standardize spotting volume, matrix composition, and acquisition parameters; consider automated spotting platforms [108,109]

**Table 6:** Different MALDI-based spectral phenotyping strategies in GBM.

Level	Approach	Outcome	Source
**Single cell**	MALDI-MSI + MALDI-IHC	Cell-type classification	[3]
**Tissue cohort**	Lipid + peptide MSI (“dry proteomics”)	Prognostic GBM clusters	[112]
**Biofluids**	MALDI-TOF MS + fractal ML	GBM vs. healthy diagnosis	[111]

## Data Availability

No new data were created or analyzed in this study. Data sharing is not applicable to this article.
